# In Vitro and In Vivo Assessments of Anti-Hyperglycemic Properties of Soybean Residue Fermented with *Rhizopus oligosporus* and *Lactiplantibacillus plantarum*

**DOI:** 10.3390/life12111716

**Published:** 2022-10-27

**Authors:** Istiqomah Hariyanto, Chia-Wen Hsieh, Yueh-Han Hsu, Lih-Geng Chen, ChiShih Chu, Brian Bor-Chun Weng

**Affiliations:** 1 Global Master Program of Life Sciences, College of Life Sciences, National Chiayi University, Chiayi 60000, Taiwan; 2 Department of Microbiology, Immunology and Biopharmaceuticals, National Chiayi University, Chiayi 600355, Taiwan; 3 Department of Internal Medicine, Division of Nephology, Ditmanson Medical Foundation Chiayi Christian Hospital, Chiayi 60002, Taiwan; 4 Department of Medical Research, China Medical University Hospital, Taichung 404327, Taiwan; 5 Department of Nursing, Min-Hwei Junior College of Health Care Management, Tainan 73658, Taiwan

**Keywords:** soybean residue, *Rhizopus oligosporus*, *Lactiplantibacillus plantarum*, isoflavone, γ-aminobutyric acid (GABA), antioxidant, hyperglycemia, kidney, histopathology, diabetic mice, solid-state fermentation, blood glucose level monitoring, glucose tolerance test, kidney histopathological examination

## Abstract

Soy isoflavones possess antioxidative, anti-inflammatory, anti-diabetic and phytoestrogenic properties. Soybean residue contains a fair amount of nutrients such as glycosylated isoflavones, minerals and dietary fibers, and is a substantial waste product produced from soymilk and tofu manufacturing. A solid-state fermentation of soybean residue by *Rhizopus oligosporus* or co-inoculated with *Lactiplantibacillus plantarum* improves the availability of isoflavones and GABA content which is attributed to ameliorated hyperglycemic symptoms in STZ-induced hyperglycemic mice. The effortless solid-state fermentation with present microbial manipulation supports an anti-hyperglycemia value-added application of soybean residue for functional food development. **Background**: Due to an awareness of the food crisis and with a rapidly rising prevalence of diabetes, recycling the substantial fibrous soybean residue disposed from soy industries has received consideration. **Methods**: *Lactiplantibacillus plantarum* was previously screened for active glutamate decarboxylase, and β-glucosidase activities were adopted for the fermenting of soybean residue using a traditional tempeh solid-state fermenting process with fungal *Rhizopus oligosporus*. Fermented soybean residue was chemically analyzed and functionally assessed in in vitro and in vivo hyperglycemic conditions. **Results**: A 48 h longer solid-state fermentation of the soybean residue co-inoculated with *R. oligosporus* and *L. plantarum* showed improved contents of isoflavone aglycones and GABA which were attributed to augmented antioxidative capacity, lowered ROS level, improved blood biochemistry, and better blood glucose homeostasis in STZ-induced hyperglycemic mice. **Conclusion**: The advantages of a food industrial effortless fermentation process, and a health nutritional endorsing anti-hyperglycemic value-added property offer a practical alternative in recycled soybean residue.

## 1. Introduction

Soy is widely consumed in Asia, approximately 5-times higher than in western countries. It is associated with a lower risk of diabetes demonstrated in numerous retrospective cohort studies. Anti-diabetic properties of soy isoflavones have been well evidenced in mechanistic actions [1]. Naturally, isoflavone exist as glycosylated conjugates by bonding in cellulosic moieties, which hinders its bioavailability. Soybean residue, also called dòuzhā in Chinese, is a primary waste by-product from soymilk and tofu manufacturing. Nutritional values are indeed still precious which contains fairly amounts of proteins, fat, isoflavones, minerals and dietary fibers. Making all-out utilization of soybean residue may bring multiple advantages in surplus profits of the soy industry, choice of ingredients in the feed and food industry, alleviating food crisis and achieving carbon neutrality.

The fermentation process is a conventional practice to preserve the soy product, eliminate anti-nutritional factors, enhance flavors and upgrade nutritional values. Particularly, soy fermentation catalyzes glycosylated moieties maximizing bioavailability of soy containing nutrients. Soybean tempeh is produced with fungal *Rhizopus* spp. such as *R. oligosporus* in a solid-state fermentation which is relatively simple and less energy consuming than submerged fermentation [2]. Mycelium growth of *Rhizopus oligosporus* helps in transforming nutritional quality by hydrolyzing protein into peptides, converting phytic acid into inorganic phosphate and degrading oligosaccharides into monosaccharides [3]. Moreover, solid-state fermentation with *R. oligosporus* promotes antioxidant capacity and content of vitamins B and C [4]. Lactic acid bacteria are commonly used inoculum known to inhibit growth of undesirable bacteria, adding aroma and flavors hence they are widely use in food preservation and development purposes. *Lactiplantibacillus plantarum* spp. are considered as one of most versatile and industrial friendly lactic acid bacteria. Recently, probiotic roles of *L. plantarum* in inhibiting difficult pathogenic Gram-negative bacteria, and modulating gut microbiota in gut-brain axis have been reported [5,6]. 

Furthermore, γ-aminobutyric acid (GABA) is synthesized as neurochemical in nervous system. The dietary source of GABA is essentially from consuming fermented food by lactic acid bacteria [7]. Peripheral GABA intake is critical in many health-related matters, the anti-diabetic therapeutic function in promoting pancreatic β-cell regeneration has recently demonstrated in vitro and in vivo [8]. The health implications of microbial GABA in diabetes, gut microbiota and digestive physiology are under intensive investigations. A study with red bean as tempeh substrate by Chen et al. [9] identified various GABA producing *Rhizopus* strains isolated from banana leaf, found their GABA producing can be constantly improved when co-culture with *L. rhamnosus*. *L. plantarum* is one of the most abundant and commonly isolate lactic acid bacteria in soymilk and tofu producing facility. Previously, an UV-mutant screened efficient GABA producing *L. plantarum* was further selected for active isoflavone deglycosylation under an arginine-penicillin negative selection [10] is implanted in current investigation. 

High prevalence in upper-middle income countries (1.5 million) and low in low-income countries (0.3 million) [11] implies diabetes mellitus is a metabolic disease highly related to food and lifestyle. Hyperglycemia is characterized with diabetes in attributing to oxidative stress, chronic inflammation, renal lesions, and cardiovascular complications. For insulin dependent diabetes, therapy could be painful due to once-daily insulin injection regimen of an intermediate or long-acting insulin, in combination with short-acting (regular) insulin [12]. Dietary manipulation by functional food maybe a strategy in to manage the problem. In addition to genistein, recent study in another primary soy isoflavone, daidzein has found exhibiting phytoestrogenic properties in the control of carcinogenesis, diabetes, osteoporosis and chronic inflammatory diseases in aging [13]. Soybean residue rich in dietary fiber as prebiotics has also been recently reviewed in implications to gut dysbiosis [14]. Soy yogurt produced with GABA producing *L. plantarum* has rescued gut normal phyla *Fermincutes* and *Bacerioidetes* from *Proteobacteria* in STZ-induced hyperglycemic mice (manuscript in preparation). Moreover, lately GABA has served as autocrine in converting pancreatic α-cell to β-cell [15]. 

Evidence provides a solid foundation in developing soybean residue into functional food. Fermenting soybean residue by co-inoculating fungal *Rhizopus oligosporus* and GABA producing lactic acid bacteria *L. plantarum* is therefore deserved for a controlled investigation to explore potential therapeutic advantages in hyperglycemic symptoms.

## 2. Materials and Methods

### 2.1. Soybean Residue Fermentation

#### 2.1.1. Sources of Soybean Residue and Microbes

Fresh soybean residue (dòuzhā) was sourced from a local Workshop Zuji Bean Tofu Factory (Chiayi, Taiwan, ROC), and immediately stored with airtight plastic bags in −20 °C walkin freezer until further processing. Ragi™ tempeh starter, a mixture of rice flour and *Rhizopus oligosporus* spores was purchased from PT. Aneka Fermentasi Industri (Bandung, Indonesia). Lactic acid bacteria *L. plantarum* *202*, a previous isolate of GABA producing strain was prepared by the Research Center for Herbal Medicine and Microbial Utilization, National Chiayi University (Chiayi, Taiwan).

#### 2.1.2. Solid-State Fermentation of Soybean Residues

Fresh soybean residue was stuffed in containers and autoclaved at 121 °C for 45 min. Following cooled down for 45 min at room temperature (25 °C). Subsequently, soy residue (SR) was inoculated with 5% *Rhizopus oligosporus* (*w/w*) and fermented for 24 h (RSF) and 48 h (RLF), or a combination by thoroughly mixing with 1% *L. plantarum 202* (*w/w*) fermented for 24 h (RLSF) and 48 h (RLLF). Fermentation process was achieved aerobically in glass Petri dish at temperature around 30–33 °C for 24 h and 48 h. Fermented soy residue was aliquot then frozen dried. Subsequently, dry powder was stored at −20 °C until further utilized in later experiments. Physical conditions included pH, temperature and moisture of fermentation were recorded accordingly with proper instruments.

### 2.2. Content Analysis

#### 2.2.1. Extraction of Isoflavones and GABA

An aliquot 0.5 g sample of different treatment groups of soybean residue was placed in falcon tube, and 10 mL of methanol or double distilled H_2_O were added in for extraction of isoflavones or GABA, respectively. After vortex stirred for 15 to 30 min to dissolve the samples, supernatant was collected by centrifugation at 2000× *g* for 10 min at 4 °C (Beckman 5810R, Krefeld, Germany). Supernatant was then filtrated with Millipore® filter paper of pore size 0.45 μm. The extract was kept in −20 °C freezer until further chromatographic analysis. 

#### 2.2.2. Analysis of Isoflavones

Method of high-performance liquid chromatography (HPLC) was performed at conditions of column: Li Chrospher 100RP-18e (4 mm × 250 mm, 5 μm), mobile phase: 0.05% TFA:CH3CN= 95:5_gradient_60:66 mm, UV detector 280 nm, temp 40 °C. The setting allowed the separation run time for 60 min in each sample to detect the isoflavones. The flow rate was 1.0 mL/min reversed-phase column, and the peak detection was at 280 nm. The various isoflavone in either soybean residues or fermented samples were identified by comparing retention times of known peaks and absorption spectra with reference standards. Identification in chromatography was further assured with added internal standards. The amount of specific content was calculated with area under curve against previous established individual chemical calibration curve.

#### 2.2.3. Analysis of GABA

The detection of γ-aminobutyric acid (GABA) by reversed-phase HPLC was used UV detection of pre-column OPA (o-phthaldehyde) (Sigma, PO657, St. Louis, MI, USA) derivatization of amino acids such as GABA. The injection volume of 50 µL of the extract sample of fermented soybean residue was analyzed. The standards and samples were analyzed with a linear gradient system of solvent A (water) and solvent B (methanol) of a gradient HPLC. Methodology was followed according to Oh et al. [16].

### 2.3. In Tube Assessment

#### 2.3.1. DPPH Assay

DPPH radical scavenging activity was performed as described previously with modifications [17]. DPPH radical scavenging ability of the soybean residue (fermented and unfermented) was performed in sample diluted with MeOH at concentrations around 3.33–3.36 mg/mL. Briefly, a 50 µL of extract was added into 1 mL of DPPH working solution. Then, the mixture was left to react in dark for 30 min. Subsequently, the changes of absorbance were detected at wavelength of 517 nm by an ELISA reader (Anthos 2010, Salzburg, Austria). DPPH radical scavenging activity was calculated as percent of control absorbance.

#### 2.3.2. ABTS Assay

The ABTS radical scavenging activity of the sample was performed according to Santos et al. [18] in a 96-well plate setting. ABTS radical scavenging activity of the soybean residue (fermented and unfermented) was prepared by samples diluted with ddH_2_O at 3.33–3.36 mg/mL concentrations. ABTS Reagent prepared by dissolving in ethanol (EtOH)with the absorbance of 0.70 (±0.02) at wavelength of 734 nm and at 30 °C. The ABTS Reagent was then kept at −20 °C for 16–18 h prior to use. An aliquot of 5 μL of each sample was added into each well. Then, each well was added with 200 μL of ABTS Reagent and mixed at 30 °C for 4 min under continuous stirring. Changes of absorbance was recorded. Ascorbic acid was used as standard to plot a calibration curve. The results were expressed as milligram of ascorbic acid per gram of extract (mg of Vitamin C/g). All samples were analyzed in triplicate.

#### 2.3.3. Fe(II) Ion Chelating Activity

Samples were prepared as described in previous ABTS assay. Fe(II) ion chelating activity was measured as described in commercial kit. Briefly, each of 0.2 mL sample was added 0.1 mM FeSO4 (0.2 mL) and 0.25 mM ferrozine (0.4 mL). Following an incubation at room temperature for 10 min, the absorbance of the mixture was recorded at wavelength of 562 nm. Chelating activity was calculated using the following formula:

Metal chelating activity = (A control – A sample)/A control × 100

A control is the absorbance of control reaction (without sample), and A sample is the absorbance in the presence of a sample extract.

### 2.4. In Vitro Assessment

#### 2.4.1. Cell Line and Cell Culture

MDCK (Madin-Darby Canine Kidney) cell line was purchased from the Bioresource Collection and Research Center, Hsinchu, Taiwan. The cell line was subculture according to the procedures of providers. Dulbecco’s Modified Eagle Medium (DMEM; Thermo Scientific, Waltham, MA, USA) supplemented with 10% fetal bovine serum, and 100 µL/mL penicillin was prepared for cell culture at 37 °C and 5% CO_2_ humidified incubator. Cell culture plastics were obtained from Corning Inc. (Corning, NY, USA). Cells were maintained for high glucose challenge assays. Briefly, the MDCK (1 × 10^6^/mL) cells were seeded in high glucose DMEM supplemented with 2 g/L sodium bicarbonate, 4.5 g/L glucose, 10 mL/L penicillin and 10% fetal bovine serum (Sigma-Aldrich, USA). MDCK cells cultured in the high glucose condition was termed HG-MDCK cells. 

#### 2.4.2. Cell Viability

The MTT method was a performed to assess cytotoxicity or cell viability. The MDCK (10^5^) cells were seeded in each well in 96-well plate overnight. The samples were added to wells with different concentrations and then they were further incubated for 24 h. At the end of incubation, 0.5 mg/L by MTT solution was then added for an additional 3 h incubation at 37 °C. Supernatant was discarded after centrifugation (Beckman, Germany) at 3000× *g*, for 10 min at room temperature. Then, 1:1 ratio of DMSO: EtOH solution was added to dissolve the formazan. Cell viability was detected by ELISA reader (Anthos 2010, Austria) at wavelength of 570 nm. The percentage of cell viability (%) was calculated by the equation: cell viability% = (Control − sample)/Control × 100%.

#### 2.4.3. Intracellular Reactive Oxygen Species Level

Intracellular reactive oxygen species (ROS) was detected by using cell membrane permeable probe dichlorofluorescin diacetate (DCFH-DA) (abcam®, Cambridge, UK). MDCK cells were seeded in the same manner as described previously. After 24 h incubation of HG-MDCK cells and experimental treatments, the cells were centrifugation (Beckman, Germany) at 300× *g*, for 10 min at room temperature to remove the supernatant. Each well was then added 100 μL of DCFH-DA at final concentration was 10 µM, and incubated for 15–30 min at 37 °C. Intracellular ROS level was determined by fluorescence microplate reader (BioTek FLx800TB, US) at excitation/emission wavelength of 480/570 nm.

### 2.5. In Vivo Assessment

#### 2.5.1. Animals and Treatment Preparations

Total 25 eight-weeks old male C57BL/6 mice were used and cared with approval protocol NCYU #1080424 by the Animal Care and Use Committee of National Chiayi University, Taiwan, ROC. The animals were housed in an alternate light/dark cycle of 12 h, a temperature of 25 ± 2 °C, and relative humidity of 60 to 70% environmental controlled room. Mice were maintained with experimental rodent diets (LabDiets No.5001) in *ad libdum* and free access to water at all times. After a 2 weeks of acclimation period, the mice were weighted and randomly assigned to four experimental groups and one house-keeping negative control group. Animals in the experimental groups were subjected for daily intraperitoneal injection of streptozotocin (STZ; dissolved in 0.1 mol/L Na-Citrate Buffer; pH = 4.4; 40 mg/kg body weight) for consecutively five days. At day 5, fasting blood glucose was determined and the blood glucoses above 250 mg/dL were determined as successfully induced hyperglycemic mice and were ready for the dietary intervention experiment. Healthy normal mice in negative control (NC) group were gavage administered ddH_2_O as house-keeping control, and those STZ-induced hyperglycemic mice were gavage fed with either ddH_2_O (STZ group), soybean residue (SR group), fermented soybean residue for short (SSRF group) or long (LSRF group) solid-state fermentation with co-inoculated fungal *Rhizopus oligosporus* and GABA producing *L. plantarum*. 

#### 2.5.2. Blood Biochemical Measurements

The blood biochemistry was measured with ROCHE Cobas Blood gas analyzer (Basel, Switzerland) with different kits including glycated hemoglobin (HbA1c), glutamic oxaloacetic transaminase (GOT), glutamic pyruvic transaminase (GPT), total bilirubin, total protein, blood urea nitrogen (BUN), total cholesterol, and triglycerides. Except HbA1c test was estimated using whole blood, other test items were determined using total 500 µL plasma. All samples analysis was performed by the Clinical diagnostics, Yow-Jiann Laboratory, Chiayi, Taiwan.

#### 2.5.3. Blood Glucose Level Monitoring and Glucose Tolerance Test

Blood samples were collected with heparinized capillary tubes via saphenous veins once every week. Blood samples were centrifuged (Hettich Mikro 20, Hettich, Germany) immediately to collect plasma. Plasma glucose level was determined by a blood glucose monitoring system (Ascensia Elite^®^XL, Bayer Co., 7697, Basel, Switzerland) with blood glucose test strips (Cat. No.1502, Bayer Co., Basel, Switzerland). Glucose tolerance test was conducted at week 3 and mice were fasted overnight prior to the test. All mice were given single dose glucose (0.5 g/kg BW) by intraperitoneal injection (i.p.). The blood sample was collected from a tail-tip-cut for a drop of blood at 0, 30, 60, 120 min after glucose administration. Blood glucose level was immediately determined with the above-mentioned monitoring system.

#### 2.5.4. Kidney Histopathological Examination

Mice were euthanized at the end-point of the experiment. Kidneys were dissected out and immediately soaked in 10% formalin for 3 days and then changed to 10% formalin with 4% formaldehyde solution for 5 h prior to paraffin embedding. After the dehydration process was completed, the sample was washed with xylene. The tissue samples were put together with the insertion clip into the soft paraffin zone of the paraffin meter and were soaked for 1 h. Vertical cross sections of paraffin-embedded kidney tissues were then de-waxed and stained with hematoxylin and eosin, according to previous methods [19]. After the staining process was completed, a stained tissue section was examined under CCD-equipped light microscopy under 40×, 200× and 400× magnifications. The integrity of renal glomerular unit and the features of chronic tubule-interstitial damage were assessed.

### 2.6. Statistics

For those in tube and in vitro data, mean values are presented. Results from the animal study were reported as mean ± standard deviation. All group data were analyzed by one-way ANOVA. Treatment comparisons were following Tukey multiple range tests (Sigma Plot 12.0). The significance level is 0.05 (α = 0.05).

## 3. Results

### 3.1. Characteristics of Fermented Soybean Residue and In Vitro Assessments

As shown in Table 1, the appeared hairy white color of soybean residue fermented with *R. oligosporus* for 24 h and 48 h were typically bright-white fungal mycelium growth, whereas slightly brown-yellowish color was observed in the RLSF group. Extend fermentation time contributed to dense hyphae and spore formation. The fruiting bodies (sporangia) are turning dark black with extended fermentation. Sporulation was accelerated in the RLLF group of the co-inoculated long (48 h) fermentation condition. This finding along with increased moisture and raised more than 10 °C temperature at 48 h when compared with those in 24 h fermentation. Moreover, the pH value of all groups was increased except the RLLF group was lower when compare with other fermented groups. The fungal fermentation had taken place earlier than the bacteria in the aerobic fermentation. Antioxidative activity determined by DPPH, ABT radicals scavenging proficiency and Fe(II) chelating activity demonstrated soybean residue fermented with either *R. oligosporus* or co-inoculated with *L. plantarum* have significantly promoted antioxidative capacity. Nevertheless, soybean residue fermented with combination of *R. oligosporus* + *L. plantarum 202* for 48 h had significantly lower the ABTS radical scavenging and ferrous ion binding activities in comparison with fermentation with only *R. oligosporus*. Apparently, the RLLF group having depleted antioxidants was a symbiotic outcome of dominant aged fungal spore (dark black color) under the effect of *L. plantarum 202*.

The catalytic action of β-glucosidase during fermentation may free polysaccharide conjugated polyphenolic constituents in attribution to higher antioxidative capacity in the fermented soybean residue groups. As shown in Table 2, isoflavones glucosides including daidzin, glycitin, and daidzin were not detectable in soybean residues (SR group) and in all other fermentation groups. Only a trace amount of aglycone, genistein was detected in soybean residue. In our previous experiment, a large quantity of isoflavone glucosides were quantitated in soymilk (data not shown) which may reflect the decant glycosylated isoflavones in the current soybean residue sample. On the other hand, the aglycones, genistein but not daidzein and glycitein was found in a trace amount in SR group, whereas fermentation has promoted the availability of daidzein and genistein. Nevertheless, extended fermentation from 24 to 48 h by *R. oligosporus* or co-inoculated with *L. plantarum 202* did not further significantly improve isoflavone aglycones contents. Finally, GABA is not detectable in soybean residue in current study, and it was not found in soymilk in our previous study. GABA is simply synthesized by microbes during fermentation. Both *R. oligosporus* and co-inoculum with *L. plantarum 202* produce GABA in fermenting soybean residue. The GABA content is increasing in a time-dependent manner. Co-inoculation with GABA producing *L. plantarum 202* has resulted in no additive effect on GABA content over the *R. oligosporus* fermented soybean residue as evidenced by comparing RSLF to RSF groups, and RLLF to RLF groups of respective 24 and 48 h fermentation time.

In vitro assessments with MDCK cells were performed under high-glucose stress (HG-MDCK cells). All treatment groups have exceeded 100% cell viability over the untreated HG-MDCK cells. Specifically, the RLLF group has the highest (*p* < 0.05) cell viability of 129.14% than the other groups. Moreover, MDCK cells under high glucose culture condition (HG-MDCK cells) have high oxidative pressure with increasing intracellular level of reactive oxygen species (ROS). Except the SR group has a 104.16% over the untreated group, the intracellular ROS levels were significantly lower (*p* < 0.05) in RSF and RLF groups, and the RLSF and RLLF were intermediates when compared with SR group. In general, soybean residue tempeh fermented with *R. oligosporus* exerts overall better protection in HG-MDCK cells than that of co-inoculated with *L. plantarum 202*. Coincidently, the outcomes of lowered intracellular ROS levels are in agreement with previous findings in antioxidative capacity (Table 1) that free radical scavenging activities were in an order of RLF > RSF > RLSF > RLLF > SR groups.

### 3.2. In Vivo Assessments of Fermented Soybean Residue Supplementation in STZ-Induced Diabetic Mice

#### 3.2.1. Improved Blood Biochemical Profile

In Table 3, The blood biochemistry of STZ group is significantly different from NC group indicates STZ -induced hyperglycemic mice after 4-weeks experiment period exhibited deleterious disorders. When the hyperglycemic mice daily gavage supplement for 4 weeks with soybean residue (SR group), short time fermented soybean residue (SSRF group), or long-time fermented soybean residue (LSRF group), the HbA1c which was measuring the level of glycated hemoglobin as a long-term hyperglycemia indicator has not improved as compared with those gavage dH_2_O only in the STZ group. Moreover, blood biochemicals including GOT, GPT, T-protein, T-bilirubin, BUN, and T-glyceride were all significantly improved in LSRF group. Among the test blood biochemical items, reduced blood total protein and increased blood urea nitrogen well exemplify glomeruli filtration dysfunction as kidney disorder in the STZ group, restored functions in LSRF group demonstrated no significant differences with normal mice in NC group. Nevertheless, some modest improvements in blood biochemical were also obtained in mice gavage soybean residue (SR group) and short time fermentation group (SSRF group).

#### 3.2.2. Ameliorated Blood Glucose Homeostasis

**Figure 1:**
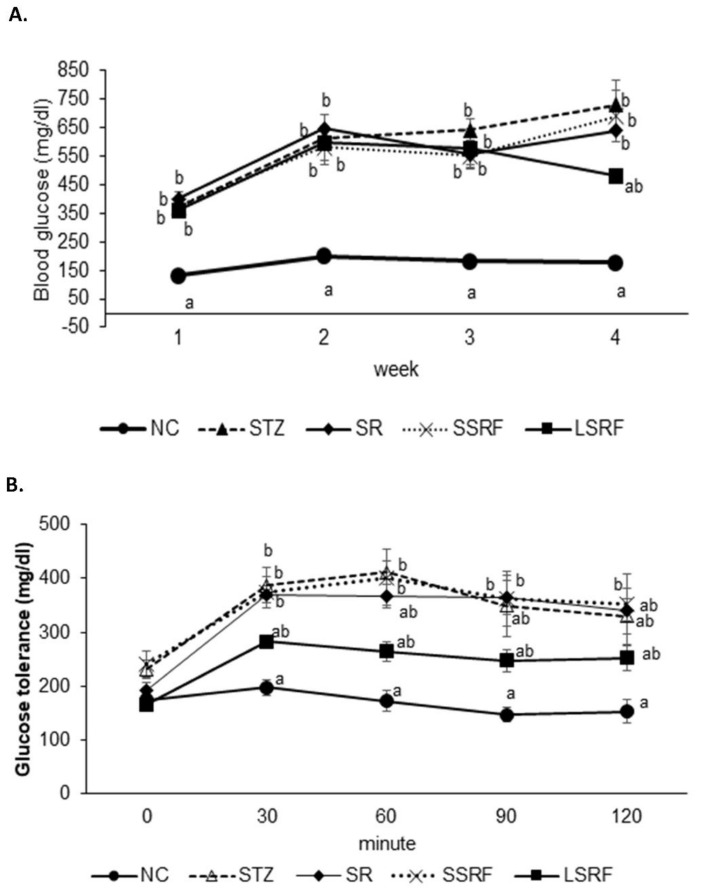
The blood glucose monitored weekly in the 4-weeks experiment (**A**). Glucose tolerance test performed at week 3 (**B**). The NC is negative control group. Additionally, the STZ-induced hyperglycemic mice are gavage fed dH2O (STZ group), soybean residue (SR group), short time soybean residue fermentation (SSRF group) and longtime soybean residue fermentation (LSRF group). The mean values indicated with letter difference is statistically different at *p*-value less than 0.05.

Results of fasting blood glucose levels monitored once every week is shown in Figure 1A. Hyperglycemic condition was obvious of the blood glucose level above 300 mg/dL at initial testing in those streptozotocin (STZ) induced hyperglycemic mice, whereas normal health mice in NC group was remaining steady around 150 mg/dL. The elevated fasting blood glucose was reduced since week 2 to week 4 in the LSRF group. The steady reduction has attributed to the statistical insignificant in comparing with the NC group at week 4. Nevertheless, the departure of blood glucose level is quite late until the last week of the experimentation in LSRF group but not SSRF group indicate soybean residue of longer fermentation is required for the improved blood homeostasis. Moreover, glucose tolerance test conducted at week 3 demonstrated single dose glucose injection had resulted in elevated blood glucose from 30 min to 120 min in the STZ-induced diabetic mice while the mice in NC group remained in a steady well-maintained blood glucose level (Figure 1B). In agree with the results in the fasting blood glucose level monitoring over time, the LSRF group exhibits improved blood glucose control in the glucose tolerance test, and SR and LSRF groups has limited effect in blood homeostasis. 

#### 3.2.3. Normalized Intracellular ROS Level in Leukocytes

**Figure 2:**
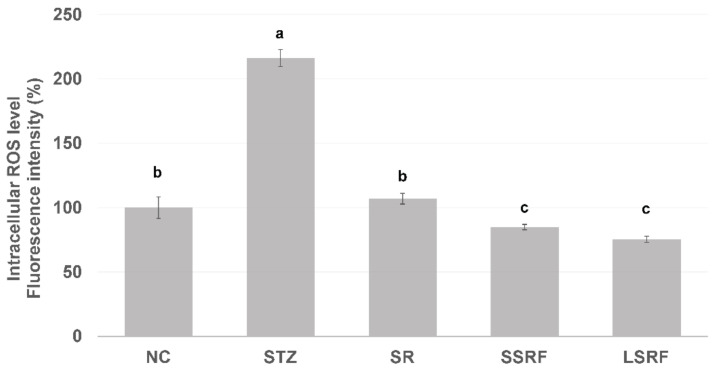
Intracellular reactive oxygen level (ROS) of leukocytes isolated from the healthy negative control group (NC), and those STZ-induced hyperglycemic mice gavage supplemented with dH2O (STZ group), soybean residue (SR group), short time soybean residue fermentation (SSRF group) and longtime soybean residue fermentation (LSRF group). The mean values indicated with letter difference is statistically different at *p*-value less than 0.05.

Increased leukocyte ROS level may represent developed inflammatory status, and it was significantly (*p* < 0.05) elevated in mice gavage dH_2_O only of the STZ group than those of NC group (Figure 2). It has shown the intracellular ROS level of SR group was not different from NC group, whereas the SSRF and LSRF groups were significantly lower (*p* < 0.05) than the STZ group as well as the NC group. The outstanding control in leukocytes intracellular ROS level by daily gavage fermented soybean residue in hyperglycemic mice indicate a well-managed chronic inflammation in hyperglycemia.

### 3.3. Protection of Hyperglycemic Mice as Evidenced by Gross Anatomy and Histopathological Evaluations in Kidney

**Figure 3:**
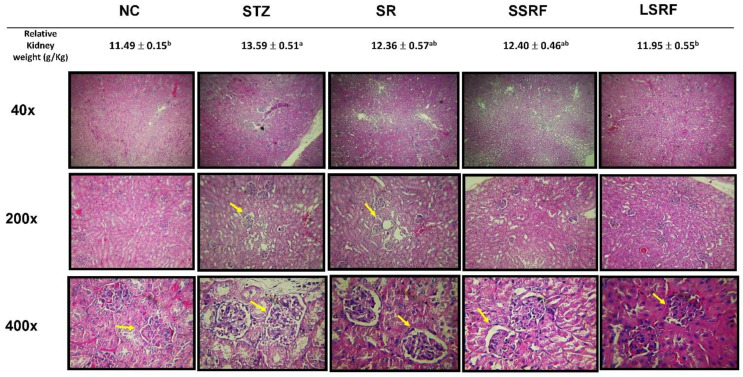
Relative kidney weight and histopathological assessments in kidney H&E stain sections under magnifications of 40×, 200×, and 400×. The yellow arrow indicates interstitial space between basal membrane and vessels in a glomerular unit. The negative control group (NC), and those STZ-induced hyperglycemic mice gavage supplemented with dH2O (STZ group), soybean residue (SR group), short time soybean residue fermentation (SSRF group) and longtime soybean residue fermentation (LSRF group).

In Figure 3, relative kidney weight (g/kg BW) and kidney tissue sections for histopathological examinations are demonstrated. Prolonged hyperglycemia causes glomeruli damages leading to kidney dysfunction. As evidenced previously with elevated leukocytes intracellular ROS level implied chronic inflammation in hyperglycemic mice, swollen kidney is confirmed by significant (*p* < 0.05) increase relative kidney weight in STZ group and microscopically exemplified extra interstitial space. The swollen size (relative kidney weight) of kidney was significantly (*p* < 0.05) diminished and nearly return back to normal size in the LSRF group. In addition, SR and SSRF groups had intermediated relative kidney weights which were insignificantly to the NC group. Nevertheless, histopathological examination of nephropathy revealed irregular edge of basal membrane of glomerular regions and expanded gap (indicated in yellow arrows) in the mesangial area of kidneys in the mice gavage dH_2_O (STZ), soybean residue (SR) and short time fermented soybean residue (SSRF) of the STZ-induced hyperglycemic mice. The more compact glomeruli and smaller gap between basal membrane and mesangial area is apparent in the LSRF group paralleled to the normal control NC group.

## 4. Discussion

Soybean is probably the most leading food grain consumed in the world. In Taiwan, estimates of at least 10 kg soybean is consumed on average per person yearly (reference from Agriculture and Food Agency, Council of Agriculture, Taiwan). Soy consumption as in the basis of soymilk and tofu has steadily increased in recent years along with higher health perspective and vegan diet in popularity. Soybean residue is major waste by-product from soymilk industry. They are mainly utilized as animal feed and fertilizer. Nutrient composition of soybean residue can be varied dramatically by the heating temperature, time and heating process performed prior or after grounding of the soaked soybeans [20,21]. At dry matter basis, soybean residue contains nutrients in average of 15–33% protein, 42–58% dietary fiber and 3–4.5% ash, while isoflavone aglycones and glucosides are 5.4% and 10.3%, respectively. In current study, isoflavones glucosides or aglycones were hardly detected in soybean residue, and only trace genistein (17 μg/g) was found. Instead isoflavones content in soymilk produced in the current study can be as high as 4.6 mg/g in dry basis (data not shown). Therefore, although current fermentation process has increased the availability of isoflavones in aglycones form, only negligible isoflavones left to be available to utilize. We compared the current soybean residue “dòuzhā” obtained from traditional Chinese soymilk and tofu manufacture to the referenced “okara” have found approximately only one tenth of total isoflavones is retained. Previous reports [22,23,24] indicate the isoflavone content in okara produced from soymilk processing retains as high as approximately 12–30% of total soybean isoflavones, and the genistin and daidzin are dominant isoflavone in okara have reported to be about 0.33 mg/g and 0.25 mg/g, respectively. Surprisingly, inconsistent isoflavones content in soybean residues imply the efficacy in optimizing utilization of soy isoflavones in soy products demand more investigation.

Soybean fermentation in a solid-state condition with *R. oligosporus* for 10 days had increased 41% or 255% of phenolic contents with water or ethanol extraction, respectively [25]. *Rhizopus oligosporus* is an efficient β-glucosidase producer during active mycelium growth, which was responsible for transforming soybean into mushed white hairy tempeh. Current experiment adopted traditional tempeh producing with an aerobic solid-state fermentation had active fungal mycelium growth as typical bright white color on soybean residue fermented with *R. oligosporus* for 24 h (RSF group) and 48 h (RLF group). When *R. oligosporus* co-inoculated with *L. plantarum 202* in a same fermentation condition, fungal mycelium growth seemed to arrest at 48 h of longer fermentation, and blackish dark appearance indicated the accelerated sporulation in generating black spores. Feng et al. [26] fermented barley-based tempeh with lactic acid bacteria species found aroma and dark color were gaining during extended fermentation time up to 24 h. In aerobic condition, fungal *R. oligosporus* fermentation can moderately raise pH value, while *L. plantarum* dampen down the rising pH during the extended fermentation. Moreover, temperature and moisture were significantly increased over time as in RLLF group as compared to the RLSF group which may further promote fungal sporulation. Therefore, soybean residue fermentation by co-inoculated with fungal *R. oligosporus* and bacterial *L. plantarum*, the lactic acid bacteria such as *L. plantarum spp.* may contribute to accelerate fungal generation in growth of mycelium and spore formation by raising temperature, reducing pH and possibly synergistically enzymatic degradation in facilitating a symbiosis. Nevertheless, extend fermentation time has also contributed to dense mycelium but darker color from promoted fungal growth and sporulation as demonstrated in the RLLF group. It has been reported lactic acid bacteria especially *L. plantarum are* actively inhibit growth of pathogenic bacteria during fermentation, and also exhibit a broad spectrum anti-fungal activity [27,28]. In current result, apparently, *R. oligosporus* is not affected by the *L. plantarum 202* previously screened with GABA producing capacity as evidenced by the dense mycelium growth in RLSF group than the RSF group. This is in agreement with Feng et al., [26] who investigated the growth of *R. oligosporus* as affected by co-inoculate with 4 different lactic acid bacteria, fungal growth as determined by ergosterol content and hyphal length was not affected by tested strains of lactic acid bacteria including *L. plantarum* among the other species.

Antioxidative capacity as measured by DPPH assay, ABTS radical scavenging activity and Ferrous ion chelating activity has significantly increased in current fermented soybean residue, which was in agree with others that *R. oligosporus* promotes antioxidant activity when fermented with soybean, and other various substrates [4,29,30]. Nevertheless, co-inoculation with *L. plantarum 202* has negatively impact on the antioxidative capacity of fermented soybean residue. In addition, extended fermentation time from 24 to 48 h has not further augment the advantage. On the other hand, we showed co-inoculate with *L. plantarum 202*, and in an extended fermentation time significantly (*p* < 0.05) lowered the antioxidative capacity of soybean residue tempeh particularly in the Fe ion chelating activity. The ferrous ion chelating emphasis on the ability of reducing electron of ion rather than stabilizing free radicals with proton donating as measured using ABTS assay. It is assumed that prolonged fermentation (48 h) could have negative impact on fungal antioxidative system. Furthermore, phytic acid presents in a fair quantity (0.5–1.2 g/100 g) in soybean residue is good antioxidant but also an anti-nutritional factor by reducing availability of essential minerals [31]. Buckle, K.A. [32] examined six traditional tempeh producing *R. oligosporus* spp. found low efficacy in reducing phytic acid. The lactic acid bacteria on the other hand are well known in reducing phytic acid by generating phytase. It has been reported that *L. plantarum* efficiently reduce anti-nutritional factors in soybean fermentation [33]. Consequently, the reduced antioxidative capacity during extended fermentation in the RLLF group maybe also attributed by reducing phytic acid content by the beneficial property of *L. plantarum* on phytase synthesis in reducing anti-nutritional factors. Hence, soybean residue fermentation by co-inoculated with *R. oligosporus* and *L. plantarum 202* under a proper fermentation time between 24 h to 48 h may enable optimal antioxidative chemical contents and maximizing depletion of harmful anti-nutritional factor.

Soybean is a rich dietary source of isoflavones, while majority of isoflavones are bonded in glucosidic moiety with low bioavailability. Historically, fermentation is a critical tactic in optimizing intake of the precious contents. A study in soybean tempeh processing variables with a mathematical modeling demonstrated 18 h fermentation has maximum bioconversion of glycosylated isoflavones into aglycones [34]. In current study, soybean residue fermented with *R. oligosporus* or co-inoculated with *L. plantarum 202* has improved the availability of isoflavones by increasing levels of isoflavone aglycones, genistein and daidzein of all fermentation groups. Extended fermentation time from 24 to 48 h has only marginally increased daidzein content but not genistein. This is partially in agree with our previous findings (in submission) in soymilk fermentation with *L. plantarum* that the daidzein is more readily available than genistein during extended fermentation. Daidzein is also preferentially uptake in mice. In the current study, GABA is a microbial secondary metabolite which has been detected in all fermentation groups but not in soybean residue (SR group). The GABA production was twice higher in 48 h fermentation than in the 24 h indicates GABA production is fermentation time dependent. Out of our expectation, there was no significant difference in GABA level between fermented soybean residue by *R. oligosporus* or co-inoculated with *L. plantarum 202*. The strain of *L. plantarum 202* adopted in current study was screened selection on efficient GABA production. GABA was only marginally higher in RLLF group than in RLF group. Additionally, the RLSF group had even a lower level than that in RSF group. The role of GABA producing *L. plantarum 202* in GABA content of soybean residue fermentation seems not assured, since *Rhizopus* species are capable of GABA synthesis [7]. Our results show GABA producing *L. plantarum* strain seems to have no synergistic effect on GABA production when co-inoculated with *R. oligosporus*. Nevertheless, growth of *L. plantarum* may increase organic acids which gradually acidify the fermentation environment. The decarboxylation of glutamate consumes intracellular proton which helps to maintain a neutral pH of cytoplasm in the low pH environment. Hence, both *L. plantarum 202* and *R. oligosporus* having high glutamate decarboxylase activity may help their stabilizing pH along with GABA synthesis in current co-inoculated fermentation treatments, which might also enable them as potential probiotics. Recently, Chen et al., [9] reported GABA content was higher in co-inoculated group with *R. oligosporus* and *L. rhamnosus* (BCRC 16000) in red bean tempeh. Moreover, although using different substrates, under a same 48 h fermentation time, their *R. oligosporus* strain and current one seems to have comparable efficacy in GABA production of 10 g/kg in red bean formation and current one in RLF group of 6.4 g/kg. Since GABA is synthesized from glutamic acid, red bean indeed is one of the high glutamine foods. Whether the available glutamic acid in soybean residue fermentation is limited the GABA content in current co-inoculation process will require further elucidation.

Streptozotocin (STZ) is highly selective pancreatic β-cell cytotoxic agent that can induced diabetic like hyperglycemic symptoms in non-obese mice [35]. STZ-induced hyperglycemic mice in SSRF and LSRF groups daily gavage soybean residue tempeh that fermented for either 24 or 48 h, respectively with co-inoculated *L. plantarum 202* have improved blood biochemical, blood glucose homeostasis, intracellular ROS levels, and glomeruli integrity of kidney. Soy genistein is known in promoting pancreatic β-cell function [1]. In addition, soy daidzein as a potent phytoestrogen exhibits beneficial property in metabolic syndrome [36]. GABA shows to depress inflammatory cytokines, suppress insulitis and promoting β-cell regeneration in both in vitro and in vivo studies [8]. Dietary supplementation with *L. plantarum 202* co-inoculated soybean residue tempeh may also exhibit pre- and probiotics properties in modulating gut microbiota.

## 5. Conclusions

When *R. oligosporus* combined with GABA producing *L. plantarum 202* in inoculating soybean residue under a solid-state fermentation have demonstrated having multiple advantages. The novel soybean residue tempeh has fortified with isoflavones aglycones and GABA which can be developed into functional food in improving glucose homeostasis in hyperglycemia. Additionally, the effortless aerobic solid-state fermentation provides a practical application in producing value-added soybean residue and recycle the waste for carbon neutral production in soy industries. Finally, a symbiosis of GABA-producing *L. plantarum* and fungal *R. oligosporus* during soybean residue fermentation is obtained, their probiotic property in modulating gut microbiota is of interest for further exploration. Microbial secondary metabolites such as GABA serving as bioactive natural compounds deserve more attentions for their health implications.

## Figures and Tables

**Table 1:** Characteristics of soybean residues fermentation and antioxidative property.

	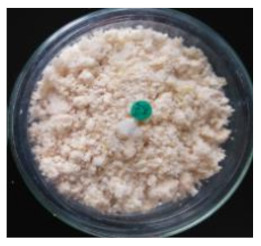	* 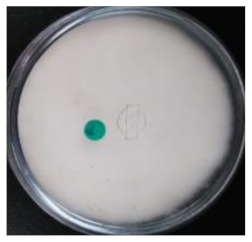 *	* 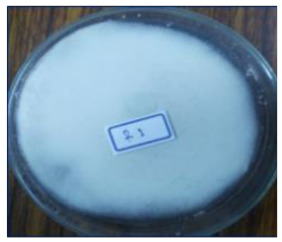 *	* 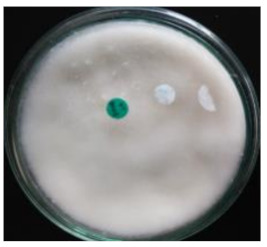 *	* 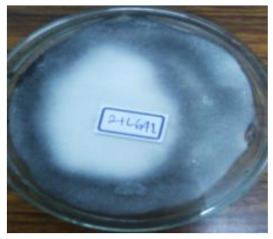 *
Fermentation Condition & Antioxidative Capacity	SR Soy Residues	RSF R. oligosporus 24 h	RLF R. oligosporus 48 h	RLSF R. oligosporus + *L. plantarum* Short Fermentation 24 h	RLLF R. oligosporus + L. plantarum Long Fermentation 48 h
Moisture	60.8 ^a^	50.4 ^c^	55.6 ^ab^	44.6 ^c^	56.5 ^ab^
Temperature	26.3 ^d^	35.7 ^c^	43.3 ^b^	35.2 ^c^	45.7 ^a^
pH	5	6	6	6	5
DPPH%	13.8 ^c^	51.9 ^a^	53.9 ^a^	47.9 ^ab^	44.3 ^b^
ABTS%	3.62 ^c^	43.1 ^a^	46.7 ^a^	14.0 ^b^	10.9 ^b^
Fe(II) Chelating%	5.86 ^d^	42.7 ^a^	44.6 ^a^	39.1 ^ab^	19.2 ^c^

The mean values indicated with letter difference is statistically different at *p*-value less than 0.05.

**Table 2:** The isoflavone and GABA contents in fermented soybean residues and their in vitro assessments.

Contents of Functional Chemicals (μg/g *w/w*)					
Item (mg/g)	SR Soy Residues	RSF R. oligosporus 24 h	RLF R. oligosporus 48 h	RLSF R. oligosporus + L. plantarum 24 h	RLLF R. oligosporus + L. plantarum 48 h
Genistin	ND	ND	ND	ND	ND
glycitin	ND	ND	ND	ND	ND
daidzin	ND	ND	ND	ND	ND
Genistein (aglycone)	17.0	20.1	20.7	22.2	19.4
Glycitein (aglycone)	ND	ND	ND	ND	ND
Daidzein (aglycone)	ND	21.4	24.5	24.6	26.8
GABA	ND	0.3154	0.6387	0.2676	0.6762
In vitro assessments on HG-MDCK cells					
Cell viability (%)	118 ^b^	121 ^b^	103 ^b^	109 ^b^	129 ^a^
Intracellular ROS (%)	104 ^a^	88.9 ^c^	87.5 ^c^	91.9 ^b^	93.2 ^b^

The mean values indicated with letter difference is statistically different at *p*-value less than 0.05. ND represent not detected.

**Table 3:** The blood biochemistry.

Items	Treatments				
	NC	STZ	SR	SSRF	LSRF
HbA1c (mg/dL)	2.9 ± 0.12 ^a^	3.8 ± 0.19 ^b^	3.9 ± 0.25 ^b^	4.2 ± 0.73 ^b^	3.8 ± 0.18 ^b^
GOT (IU/L)	41 ± 5 ^a^	177 ± 14 ^b^	96.6 ± 6 ^c^	53.4 ± 5 ^c^	61.6 ± 10 ^c^
GPT (IU/L)	15.4 ± 3.9 ^a^	89 ± 12 ^b^	33.6 ± 3.7 ^ab^	26.6 ± 3.9 ^ac^	38 ± 7.3 ^c^
T-Protein (IU/L)	1.7 ± 0.3 ^a^	1.3 ± 0.1 ^b^	1.5 ± 0.08 ^ab^	1.5 ± 0.17 ^ab^	1.6 ± 0.11 ^ac^
T-Bilirubin (IU/L)	0.208 ± 0.01 ^a^	0.244 ± 0.01 ^b^	0.226 ± 0.01 ^ab^	0.218 ± 0.01 ^ac^	0.214 ± 0.01 ^ac^
BUN (IU/L)	6.3 ± 0.60 ^a^	8.1 ± 0.70 ^b^	6.6 ± 0.60 ^ac^	6.5 ± 0.17 ^ac^	6.4 ± 0.30 ^ac^
T-Cholesterol (IU/L)	22.4 ± 5 ^a^	30.2 ± 2.3 ^b^	24.8 ± 2.5 ^ab^	24.4 ± 0.5 ^a^	25 ± 0.7 ^ab^
T-Glyceride (IU/L)	8.4 ± 1.8 ^a^	20 ± 4.8 ^b^	16.2 ± 3.1 ^bc^	13.8 ± 2.6 ^ac^	10.4 ± 1.3 ^c^

The items analyzed including HbA1c (glycated hemoglobin), GOT (glutamic oxaloacetic transaminase), GPT (glutamic pyruvic transaminase), T-protein (total protein), T-bilirubin (total bilirubin), BUN (blood urea nitrogen), T-cholesterol (total cholesterol), and T-glyceride (total glyceride). Treatment groups are NC (healthy housekeeping negative control), SR (soybean residue), SSRF (short time soybean residue fermentation), LSRF (long time soybean residue fermentation). The data presented as mean ± SD and letter difference across treatment groups is statistically different at *p*-value less than 0.05.

## Data Availability

The data presented in this study are available on request from the corresponding author.
